# KChIP2 regulates the cardiac Ca^2+^ transient and myocyte contractility by targeting ryanodine receptor activity

**DOI:** 10.1371/journal.pone.0175221

**Published:** 2017-04-06

**Authors:** Drew M. Nassal, Xiaoping Wan, Haiyan Liu, Kenneth R. Laurita, Isabelle Deschênes

**Affiliations:** 1 Heart and Vascular Research Center, Department of Medicine, MetroHealth Campus, Case Western Reserve University, Cleveland, Ohio, United States of America; 2 Department of Physiology and Biophysics, Case Western Reserve University, Cleveland, Ohio, United States of America; New York Medical College, UNITED STATES

## Abstract

Pathologic electrical remodeling and attenuated cardiac contractility are featured characteristics of heart failure. Coinciding with these remodeling events is a loss of the K^+^ channel interacting protein, KChIP2. While, KChIP2 enhances the expression and stability of the Kv4 family of potassium channels, leading to a more pronounced transient outward K^+^ current, *I*_to,f_, the guinea pig myocardium is unique in that Kv4 expression is absent, while KChIP2 expression is preserved, suggesting alternative consequences to KChIP2 loss. Therefore, KChIP2 was acutely silenced in isolated guinea pig myocytes, which led to significant reductions in the Ca^2+^ transient amplitude and prolongation of the transient duration. This change was reinforced by a decline in sarcomeric shortening. Notably, these results were unexpected when considering previous observations showing enhanced *I*_*Ca*,*L*_ and prolonged action potential duration following KChIP2 loss, suggesting a disruption of fundamental Ca^2+^ handling proteins. Evaluation of SERCA2a, phospholamban, RyR, and sodium calcium exchanger identified no change in protein expression. However, assessment of Ca^2+^ spark activity showed reduced spark frequency and prolonged Ca^2+^ decay following KChIP2 loss, suggesting an altered state of RyR activity. These changes were associated with a delocalization of the ryanodine receptor activator, presenilin, away from sarcomeric banding to more diffuse distribution, suggesting that RyR open probability are a target of KChIP2 loss mediated by a dissociation of presenilin. Typically, prolonged action potential duration and enhanced Ca^2+^ entry would augment cardiac contractility, but here we see KChIP2 fundamentally disrupts Ca^2+^ release events and compromises myocyte contraction. This novel role targeting presenilin localization and RyR activity reveals a significance for KChIP2 loss that reflects adverse remodeling observed in cardiac disease settings.

## Introduction

The development of heart failure, whether from atrial fibrillation, hypertrophy, or myocardial infarction, culminates in compromised contractility and the insufficient ability to pump blood for the demands of the body. While current therapeutics have done much to stem and treat the progression of heart failure, we are still unable to stop the progression, let alone reliably reverse adverse remodeling. Given the ever growing rise in number of heart failure patients, the need for greater understanding of the molecular targets for disease onset and progression are required.

The potassium channel interacting protein 2 (KChIP2) is a protein that consistently experiences degradation and sustained loss early in hypertrophy and heart failure [1, 2]. This reproducibility suggests its loss may not just be symptomatic, but relevant to the progression and ailments of heart failure. KChIP2 is well-established as an accessory subunit and modulator of the Kv4 family of potassium channels, responsible for encoding the fast transient outward potassium current, *I*_to,f_, critical for early phase 1 repolarization during the cardiac action potential [3]. This current serves as the primary repolarizing current in rodents, directly contributing to action potential duration (APD), whereas in larger mammals it establishes the membrane potential for Ca^2+^ entry and indirectly influences APD. Through interaction with the N-terminus of Kv4, KChIP2 can lead to the enhanced trafficking and stability of these channels [4], while also enhancing the duration of channel opening and recovery from inactivation, resulting in more pronounced *I*_to,f_ [3, 5]. With the loss of KChIP2, there is an associated loss of Kv4, and the disappearance of *I*_to,f_. However, this loss in *I*_to,f_ does not appear to be a precipitating event in heart failure, as animal models with the Kv4 gene removed and resulting APD prolongation, do not display signs of heart failure remodeling [6].

Supporting the prospect of additional roles for KChIP2 that may be relevant for cardiac disease remodeling is the guinea pig myocardium, which expresses KChIP2 but not Kv4 [7, 8]. We previously showed that acute loss of KChIP2 in guinea pig myocytes led to prolonged action potential duration (APD) through increased L-type Ca^2+^ current (*I*_Ca,L_) density [8]. Indeed, we and others show that KChIP2 loss yields increased Cav1.2 protein expression [8, 9], which in the mouse, resulted in diminished *I*_Ca,L_ density due to a secondary interaction with an N-terminal fragment not conserved in the guinea pig. Additionally, KChIP2 appears to interact with Nav1.5, the Na^+^-channel alpha subunit, as part of a macromolecular interaction that includes Kv4 [10]. Beyond these secondary roles for KChIP2, other members of the KChIP family (KChIPs 1–4), which are absent from the myocardium, have more diverse roles ranging from transcriptional regulation [11, 12] to the interaction and modulation of presenilin [13, 14], a protein responsible for amyloid beta processing in the brain and more recently implicated in the potentiation of Ca^2+^ release from ryanodine receptors (RyR) [15–17]. However, these roles have not been investigated for KChIP2, the only KChIP member expressed in the heart.

Given that we previously showed acute silencing of KChIP2 in guinea pig myocytes led to enhanced *I*_Ca,L_ and APD prolongation [8], paired with the consistent KChIP2 loss observed in cardiac pathologies, we wanted to evaluate what influence acute silencing of KChIP2 has on Ca^2+^ handling and contractility. Unexpectedly, Ca^2+^ transients were reduced following KChIP2 knock-down, which further led to compromised contractile performance in the myocytes. Moreover, the attenuated Ca^2+^ release was associated with a significant decline in RyR activity. While no changes were observed in the expression of Ca^2+^ handling proteins, there was significant redistribution of the RyR modulator, presenilin, from repetitive sarcomeric localization to more disorganized and diffuse expression, potentially disrupting normal RyR Ca^2+^ release. Much like the neuronal KChIP isoforms, this suggests KChIP2 can modulate presenilin, which in turn regulates RyR activity, illustrating a novel role for KChIP2 in cardiac disease.

## Materials and methods

### Guinea pig ventricular myocytes: Isolation and short-term culture

Single ventricular myocytes were isolated from adult guinea pigs as described previously [18]. Briefly, guinea pigs were anesthetized by injection of fatal plus. Hearts were quickly removed and perfused via the aorta with a physiological salt solution (PSS) containing (in mmol/L) NaCl 140, KCl 5.4, MgCl2 2.5, CaCl2 1.5, glucose 11, and HEPES 5.5 (pH 7.4). After 5 minutes, perfusate was switched to a nominally calcium-free PSS with collagenase (Roche, 0.5 mg/mL) being added after an additional 5 minutes. After 15–20 minutes of digestion, hearts were perfused with a high K+ solution containing (in mmol/L) potassium glutamate 110, KH2PO4 10, KCl 25, MgSO4 2, taurine 20, creatine 5, EGTA 0.5, glucose 20, and HEPES 5 (pH 7.4). Ventricles were minced in high K+ solution, and single myocytes were obtained by filtering through a 115-μm nylon mesh. Myocytes were left to settle for 2 hours at room temperature before being collected in a low-speed spin. Cell pellets were resuspended in M199 medium supplemented with antibiotic and plated on uncoated 10 cm dishes. Cultures were treated with GFP adenovirus or adenovirus with a kcnip2 (KChIP2) mRNA antisense coding sequence in IRES with GFP. Cultures were then incubated in 5%CO2 at 37°C for 24 hrs.

### Viral constructs

Control GFP and KChIP2 antisense adenoviruses were used as previously described [8]. cDNA of the identified guinea pig KChIP2 antisense sequence was created and cloned into an adenoviral vector under the regulation of a CMV promoter (KChIP2-KD). The vector coexpressed GFP through an internal ribosomal entry site. A control adenoviral vector was used that omitted the antisense sequence and expressed GFP alone.

### Calcium transients, SR Ca^2+^ load, and contractions in guinea pig myocytes

Myocytes were bathed in a chamber with Tyrode’s solution composed of (mmol/L) NaCl 137, KCl 5.4, CaCl2 2, MgSO4 1, Glucose 10, HEPES 10, pH to 7.35 with NaOH. Intracellular Ca2+ transient and sarcomere shortenings were initiated by field stimulation at 2Hz at 35^°^C. Ca^2+^ transient was measured using the fluorescent Ca^2+^ indicator indo-1_AM_ as described previously [19]. Cells were loaded with indo-1AM by incubating them in Tyrode containing indo-1_AM_ (2uM) (Molecular Probes) and 0.025% (wt/wt) Pluronic F-127 (Molecular Probes) for 20 min at room temperature. The indicator was excited at 350 nm and the emitted signals were measured simultaneously at 405 nm and 485 nm. The emission field was restricted to a single cell with the aid of an adjustable window. The background fluorescence recorded from a cell without indicator loaded at both wavelengths was subtracted from the signal recorded from the cell before the fluorescence ratio was calculated. The 405nm/485nm fluorescence ratio was used to monitor changes in [Ca^2+^]_i_ produced by stimulation. Ca^2+^ transient parameters were defined referring to the methods described previously [20]. Diastolic Ca^2+^ was defined as cytosolic Ca^2+^ level just prior to the onset of the Ca^2+^ transient or just prior to the action potential upstroke in the cases where there was no obvious Ca^2+^ transient. Amplitude of intracellular Ca^2+^ transient was calculated from the difference between peak and diastolic Ca^2+^. Time to peak was measured from the onset to the peak of Ca^2+^ transient. The duration of intracellular Ca^2+^ transient was measured as the onset of the Ca^2+^ transient to the point of time when the transient decayed by 50%. SR content was compared between groups of myocytes after 10 mM Caffeine pulse induced SR Ca release using high speed solution exchange system. Sarcomere shortening was assessed using a video-based sarcomere length detection system (IonOptix Corporation) [21]. Data acquisition was operated with an Axopatch 200B patch clamp amplifier controlled by a personal computer using a Digidata 1200 acquisition board driven by pCLAMP 7.0 software (Axon Instruments, Foster City, CA).

### Immunoblotting

Freshly isolated guinea pig ventricular myocytes were cultured for 24 h at 37°C in M199 medium under control GFP or with KChIP2 antisense virus. Upon collection, cardiomyocytes were washed in ice-cold PBS and resuspended in a RIPA lysis buffer (150 mM sodium chloride, 1.0% NP-40 or Triton X-100, 0.5% sodium deoxycholate, 0.1% SDS (sodium dodecyl sulphate), 50 mM Tris, pH 8.0, plus Roche Inhibitor tablet) and then sonicated on ice to disrupt cell membranes. 30–40 μg of whole cell extract was loaded into SDS-PAGE gels, transferred to nitrocellulose membrane, and western blotting performed using antibodies against RyR (1:800, Affinity Bioreagents), Serca2a (1:1000, Dr. Periasamy, Ohio State University), Phospholamban (1:1000, Santa Cruz), NCX (1:1000, Swant), GAPDH (1:4000, Santa-Cruz), beta-actin (1:1000, Sigma-Aldrich), pan-cadherin (1:1000, Cell Signaling), and presenilin 1 (1:500, Assay Biotech). Protein concentrations were determined by the BCA method (Pierce).

### Ca^2+^ spark recordings

Line scan Ca^2+^ transients were recorded by confocal line scanning (Leica DMi8). Myocytes were preloaded with 10 μM Rhod-3 dye (Thermofisher) in a tyrode solution consisting of (mmol/L) NaCl 137, KCl 5.4, CaCl2 2, MgSO4 1, Glucose 10, HEPES 10, pH to 7.35 with NaOH and 0.1% Pluronic® F-127 (Thermofisher). Mycoytes were paced at 1 Hz for 10 beats and then the pacing was halted to record Ca^2+^ spark activity at rest. Cells were scanned longitudinally along the long axis of the myocyte. Line scan images were processed and analyzed with ImageJ software utilizing the SparkMaster plugin [22].

### Immunohistochemistry and image quantitation

Isolated guinea pig myocytes were plated on laminin coated coverslips for 2 hrs, after which the media was changed with fresh M-199 and treated with Ad.GFP or Ad.KChIP2 KD for 24 hrs. Cells were fixed in 4% formaldehyde diluted in PBS for 15 minutes. Cells were permeabilized for 10 min in PBS + 0.03% Triton X-100 and blocked for 2 hrs in a solution of PBS, 5% normal goat serum, and 1% BSA. Cells were incubated overnight with primary antibody (Presenilin 1, Assay Biotech at 1:50 and RyR, 1:50 Affinity Bioreagents) in PBS with 2% normal goat serum and 1% BSA. Cells were rinsed 3x in PBS then incubated with secondary antibody (Alexa-568 1:1000 against Presenilin 1 and Alexa-647 1:500 against RyR) in PBS with 2% normal goat serum and 1% BSA for 2 hrs at room temperature. Labeled cardiomyocytes were scanned with a Leica DMi8 confocal microscope. Fluorescence intensity profiles were generated by ImageJ using the plot profile function for the declared regions of interest. A single region of interest was evaluated per myocyte. The peaks of all resulting plot profiles within each treatment group were aligned to create a representative average trace.

### Statistical analysis

The experimental data were expressed as mean ± SEM. A Student’s t-test or a paired Student’s t-test in the case of immunoblot analysis was performed. The paired test was applied given that each set of Ad.GFP or Ad.KChIP2 KD myocytes were derived from the same cell isolation. All tests were two-sided and a significance level of p < 0.05 was defined as statistically significant (SPSS 18.0 software, SPSS, Chicago, IL).

### Ethics statement and tissue acquisition

This study was carried out in strict accordance with the recommendations in the Guide for the Care and Use of Laboratory Animals of the National Institutes of Health. The protocol for tissue isolation from adult guinea pig (Protocol Number: 2012–0175) samples was approved by the Committee on the Ethics of Animal Experiments of Case Western Reserve University. 14 male Hartley guinea pigs 5–6 weeks old from Charles River were used to conduct our studies.

## Results

**Fig 1:**
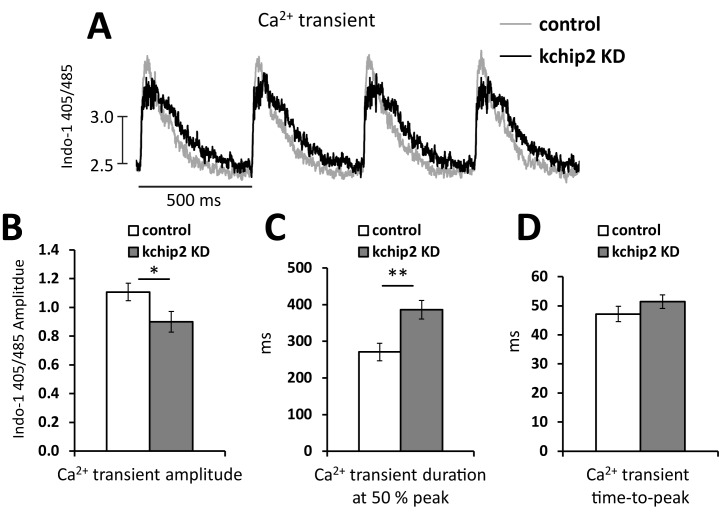
KChIP2 knock down reduces Ca^2+^ transient amplitude. **(A)** Representative Ca^2+^ transient recordings taken from isolated adult guinea pig myocytes loaded with Indo-1 and paced at a 500 ms cycle length. Recordings were taken following 24 hrs treatment with an adenovirus encoding GFP (control, n = 17) or an mRNA antisense sequence for KChIP2 (Ad.KChIP2 KD, n = 17). Summary data for the **(B)** Ca^2+^ transient amplitude, **(C)** Ca^2+^ transient duration at 50% peak amplitude, and **(D)** the transient time-to-peak. Data presented as mean ± SEM; *P < 0.05, **P < 0.01; two-tailed Student’s *t*-test.

We isolated adult guinea pig ventricular myocytes, which were subsequently treated with a control virus encoding GFP (control) or an antisense sequence for KChIP2 (KChIP2 KD) to acutely suppress expression as previously confirmed [8]. Following 24 hrs incubation, we evaluated the cells for changes to Ca^2+^ transients and contractility. In myocytes treated with KChIP2 KD we observed a significant reduction in Ca^2+^ transient amplitude by 18.8% (Ca^2+^ transient amplitude: control 1.11 ± 0.06 vs KChIP2 KD 0.90 ± 0.07 AU) (Fig 1A and 1B) at 0.5 Hz field stimulation. This change was accompanied by a significant prolongation of the Ca^2+^ transient duration (Fig 1C) but no change in the time-to-peak (Fig 1D). Remarkably, this reduction in Ca^2+^ transient amplitude and preservation of SR Ca^2+^ content occurred despite the presence of an increase in *I*_Ca,L_ [8], suggesting a potential impact of KChIP2 on calcium induced calcium release (CICR).

**Fig 2:**
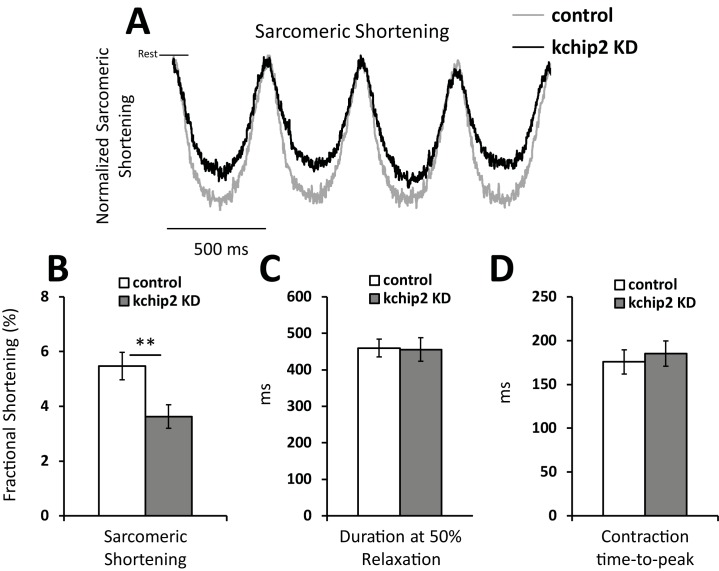
KChIP2 knock down reduces myocyte contractility. **(A)** Representative tracing of the change in distance between two consecutive sarcomeres during contraction. Cells were paced at a 500 ms cycle length for Ad.GFP (n = 26) or Ad.KChIP KD (n = 28) treated myocytes. Tracings were normalized to control cells. Summary data for **(B)** fractional shortening, **(C)** contraction duration at 50% amplitude, and **(D)** the time to peak contraction. Data presented as mean ± SEM; *P < 0.05, **P < 0.01; two-tailed Student’s *t*-test.

**Fig 3:**
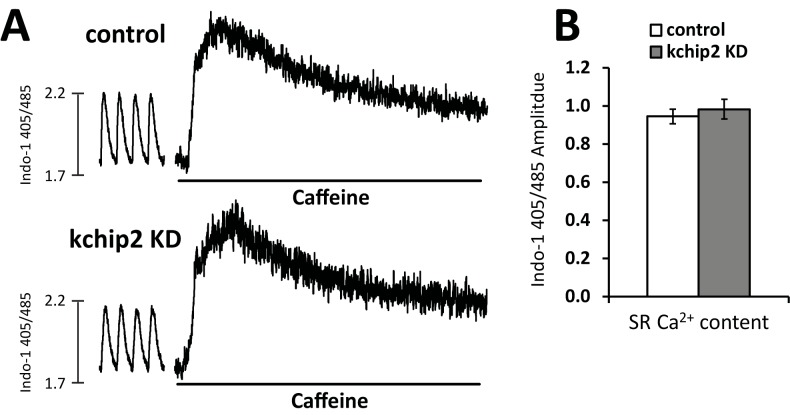
Reduced Ca^2+^ transients following KChIP2 KD are not associated with reduced SR Ca^2+^ load. **(A)** Examples of caffeine induced Ca^2+^ transients measured in cells loaded with Indo-1. Ca^2+^ transients preceding the caffeine trace were not collected in the same capture as the caffeine induced transient but were assessed with the same standard curve used in the transient recordings. **(B)** Summary data for the amplitude of caffeine induced Ca^2+^ transients for Ad.GFP (n = 13) and Ad.KChIP2 KD (n = 13). Data presented as mean ± SEM.

Coinciding with the reduction in calcium release, myocyte contractility was also compromised. With KChIP2 loss we observed a decrease in fractional shortening from 5.47 ± 0.50 in control cells to 3.63 ± 0.44% in KChIP2 KD (Fig 2A and 2B). No changes were seen to the duration or the rate of contraction between treatment groups (Fig 2C and 2D). Yet, we clearly see that with KChIP2 loss, Ca^2+^ release and contraction are weakened. Notably, SR Ca^2+^ content was assessed by caffeine stimulation showing no change between treatment groups (Fig 3A and 3B), indicating the reduction in transient amplitude is not from a loss in SR Ca^2+^.

**Fig 4:**
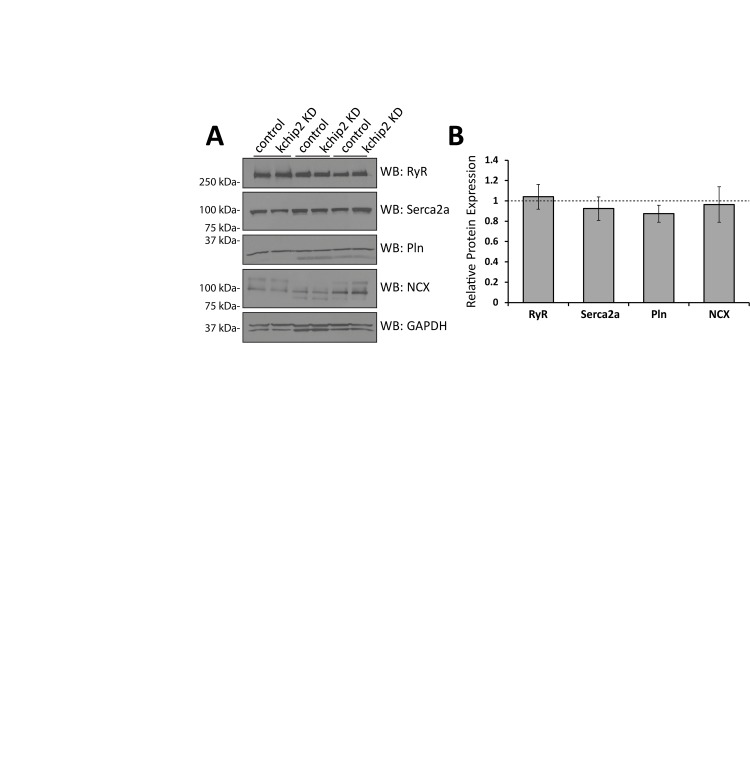
KChIP2 KD is not associated with a change in expression in the major Ca^2+^ handling proteins. **(A)** Representative immunoblots from guinea pig myocyte whole cell lysates for Ryanodine receptor (RyR), Serca2a, Phospholamban (Pln), Sodium Calcium Exchanger (NCX), and glyceraldehyde-3-phospohate (GAPDH) following treatment with Ad.GFP or Ad.KChIP2 KD for 24 hrs. **(B**) Summary data for the densitometry fold change between paired samples of each protein target normalized to GAPDH or beta-actin in Ad.KChIP2 KD (n = 3–5) treated cells compared to Ad.GFP (n = 3–5). There are no significant differences between treatment groups. Data presented as mean ± SEM.

To address how an increase in *I*_Ca,L_ could lead to suppressed Ca^2+^ transients and reduced contractility, we evaluated the canonical players in Ca^2+^ handling machinery. Ryanodine receptor (RyR2), SR Ca^2+^ ATPase (SERCA2a), phospholamban, and sodium calcium exchanger (NCX) expression, however, were all unchanged in response to KChIP2 KD (Fig 4A and 4B). The observation of no change in the SR Ca^2+^ content (Fig 3) is consistent with a lack of change in SERCA2a and phospholamban expression and regulation. While we did observe a significant prolongation of the Ca^2+^ transient, it does not appear that Ca^2+^ reuptake is responsible. Therefore, we next determined if Ca^2+^ release was instead affected by the loss in KChIP2.

**Fig 5:**
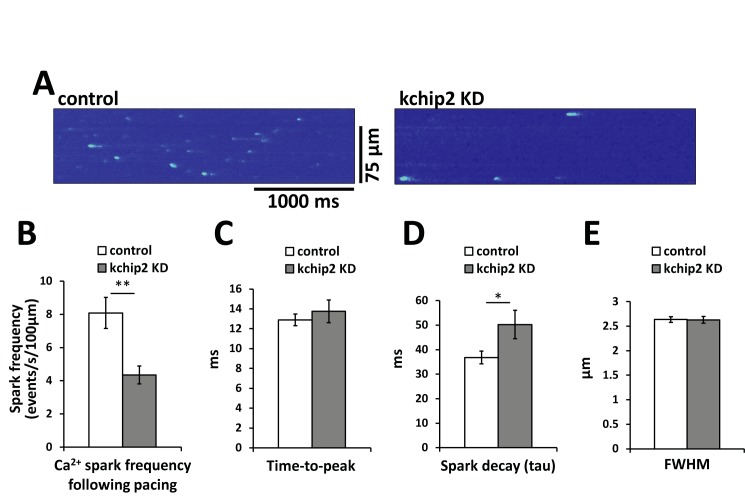
KChIP2 KD results in a significant reduction in spark frequency. **(A)** Left panel (Ad.GFP) and right panel (Ad.KChIP KD) show representative recordings of confocal line scan imaging evaluating spontaneous Ca^2+^ spark activity following 1 Hz field stimulation. Summary data between control (n = 47 cells) and KChIP KD (n = 44 cells) for the **(B)** spark frequency, **(C)** Ca^2+^ spark time-to-peak, **(D)** decay time of the Ca^2+^ spark, and **(E)** full width at half maximum (FWHM) amplitude.

While we saw no change in the amount of RyR expression, we wanted to evaluate a possible change in its function. To address this, we performed confocal line scan imaging to determine Ca^2+^ spark activity and, thus, assess RyR activity. Cells were paced for 10 beats at 1 Hz, immediately after which measurements for spontaneous Ca^2+^ release events were taken. We observed that cells with KChIP2 KD had a spark frequency 46.2% lower than control cells (Fig 5A and 5B), suggesting reduced open probability for RyR receptor in myocytes without KChIP2. Notably, this reduced activity of Ca^2+^ release can explain the reduced Ca^2+^ transient amplitude and resulting contraction events. Additionally, no change in the time-to-peak (Fig 5C) but a significant prolongation of spark decay (Fig 5D) following KChIP2 KD are consistent with the conditions observed for the overall Ca^2+^ transient. There was also no change in the full width at half maximum amplitude between treatment groups (Fig 5E), suggesting preservation of RyR clustering.

**Fig 6:**
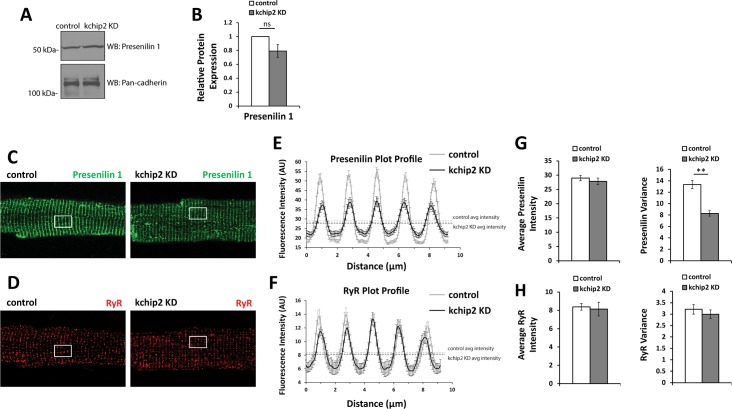
KChIP2 KD associates with a relocalization of presenilin 1 away from sarcomeric structures. **(A)** Representative immunoblot for presenilin 1 protein from whole cell lysates for Ad.GFP and Ad.KChIP2 KD treated myocytes. Protein expression was normalized to pan-cadherin expression. **(B)** Summary data for the average fold change in KChIP2 KD (n = 4) from control (n = 4) showing no reduction in presenilin 1 protein. **(C)** Representative immunostaining for presenilin 1 protein in Ad.GFP (left panel) and Ad.KChIP2 KD (right panel). Control cells show a strong sarcomeric pattern of expression that is disrupted in KChIP2 KD treated cells. White box represents region of interest selected for evaluating fluorescence intensity in ImageJ. **(D)** Representative immunostaining for RyR between Ad.GFP (left panel) and Ad.KChIP2 KD (right panel), showing preserved distribution. **(E)** Average plot profile for presenilin 1 from regions of interest (white boxes) showing the decreased peak intensity at sarcomeres and increased intensity between sarcomeres in Ad.KChIP2 KD (n = 25) compared to Ad.GFP (n = 25) myocytes. Plot profiles were created by the plot profile function within ImageJ. **(F)** Average plot profile for RyR in the same regions of interest assessed for presenilin 1, shows no change in distribution. **(G)** Summary data for the average staining intensity for presenilin 1 (left panel) and the variance (standard deviation) in the plot profile (right panel) in the regions of interest. Preservation of the average intensity reflects no change in the amount of presenilin protein, however, a decrease in variance reflects the loss in organization for presenilin 1 protein expression upon KChIP2 KD. **(H)** The same parameters used to evaluate presenilin 1 distribution but now for RyR, show no change in average intensity or heterogeneity, reflecting no change in RyR localization.

We were next curious how a loss in KChIP2 could lead to a modulated state of RyR activity. Recent investigations into the protein presenilin have shown its ability to modulate Ca^2+^ release activity through RyR [15–17]. At the same time, KChIP3 was previously discovered to regulate presenilin activity and influence the degree of regulation it has on RyR [23]. Therefore, we sought to determine if KChIP2 loss could impact presenilin expression, and thereby RyR activity. To this effect, we saw that KChIP2 KD led to no change in the amount of presenilin protein (Fig 6A and 6B). However, evaluation of presenilin localization by immunocytochemistry revealed significant reorganization of expression (Fig 6C and 6D). While control cells showed strong pesenilin alignment with sarcomeric structure, KChIP2 KD led to significant disruption of this banded pattern. Presenilin became dispersed away from sarcomeric bands towards a more unstructured state. This observation was quantitated by evaluating regions of interest of the fixed myocytes using the plot profile function within ImageJ software [24]. This revealed a reduction in peak signal intensity and an increase in minimum signal intensity following KChIP2 loss, depicting a decreased heterogeneity in the plot profile expression for presenilin (Fig 6E). At the same time, the average fluorescence intensity for the regions of interest remained unchanged, reflecting the preserved protein expression shown by immunoblot (Fig 6G). Notably, this redistribution was specific to presenilin, as RyR staining remained unchanged between treatment groups (Fig 6D, 6F and 6H). As presenilin has previously been observed to enhance the degree of Ca^2+^ release through RyR, this relocalization of presenilin away from sarcomeric structure suggests a removal of this augmented state, yielding a compromised state of Ca^2+^ release and contraction following KChIP2 loss.

## Discussion

In the present study we sought to determine the role of KChIP2 in Ca^2+^ handling and contractility in the guinea pig, particularly given our previous findings of enhanced *I*_Ca,L_ following acute KChIP2 loss [8]. Despite this observation, we found that with the same loss in KChIP2, the Ca^2+^ transient amplitude was diminished, and with it came an attenuated state of myocyte contractility. Notably, these changes occurred without an altered state of SR Ca^2+^ content or protein expression of the canonical Ca^2+^ handlers in the sarcolemma space. Instead, we observed reduced activity of the ryanodine receptor, coinciding with relocalization of presenilin, an established activator of RyR activity. Together, this implicates the loss of KChIP2 as a potential mediator of compromised cardiac contractility.

The shape and regulation of the cardiac action potential is intimately associated with the initiation and coordination of Ca^2+^ release events. In particular, the transient outward potassium current, *I*_to_, coordinates both the magnitude and duration over which Ca^2+^ can enter the cell, influencing both SR Ca^2+^ load and Ca^2+^ priming for CICR [25]. However, the guinea pig does not encode *I*_to_ yet KChIP2 expression is maintained, suggesting that any potential influence KChIP2 might have over Ca^2+^ handling and contractility would have to come independently from its established regulation over *I*_to_. To this effect, we saw that KChIP2 silencing led to a significant increase in *I*_Ca,L_ through increases in Cav1.2 protein expression, producing a significant prolongation of the action potential [8]. Given that enhanced *I*_*Ca*,*L*_ would provide more activating Ca^2+^ in the CICR response, we anticipated this phenotype would have augmented CICR and contractility. However, we instead observed a dramatic loss in Ca^2+^ transient amplitude (Fig 1A and 1B) and cell shortening (Fig 2A and 2B), indicating a fundamental disruption to the normal CICR events. As there were no changes in the expression of canonical Ca^2+^ handling proteins (Fig 4), or to SR Ca^2+^ content (Fig 3) to explain the smaller transient amplitude, this suggested a modulation to RyR activity. Such an effect was further supported by the observed reduction in Ca^2+^spark frequency following KChIP2 KD (Fig 5A and 5B).

Precedence for the ability of KChIP to modulate Ca^2+^ release events independent of its contributions to *I*_to_ and APD have previously been observed, only not in the heart. KChIP3, also known as calsenilin, another member of the KChIP family with significant expression in the brain but absent in the heart, was previously identified to bind to and modulate a class of proteins known as presenilins [13, 14]. These transmembrane proteins have garnered much attention due to mutations that have closely associated with the development of Alzheimer’s Disease. Serving as part of a γ-secretase complex, these proteins can become deregulated leading to the formation of neurotoxic amyloid-beta peptides [26]. Notably, these same mutations to presenilin 1 are strongly associated with alterations to cytosolic Ca^2+^ concentrations, [26–28] which is believed to contribute directly to disease pathogenensis. In line with this, presenilin 1 has been shown to bind to several proteins critical to Ca^2+^ release mechanisms, including the inositol 1,4,5-trisphosphate receptor (IP_3_R) [29], the *N*-methyl-D-aspartate (NMDA) receptor [30], and most relevant to our investigation, the RyR receptor [15–17]. As a consequence of its interaction with the RyR receptor, presenilin 1 enhances RyR open probability and mean Ca^2+^ current. Moreover, these gain of function mutations to presenilin 1 are shown to enhance the amplitude of Ca^2+^ release through RyR, and have also been associated with the development of dilated cardiomyopathy and heart failure [31]. Together, this suggests that loss of presenilin 1 at sarcomeric structures would lead to reduced RyR activity and thereby Ca^2+^ release, as we see in our investigation.

Notably, KChIP2 shares a high degree of sequence homology with KChIP3/calsenilin, further supporting that its ability to modulate presenilins is a conserved function. Indeed, KChIP3 is capable of modulating Kv4 channel expression and kinetics in the same manner as KChIP2 [5, 32], and we have shown that KChIP2 much like KChIP3/DREAM can act as a transcriptional repressor [33], reinforcing the notion of shared roles between these family of proteins. Therefore, while KChIP3 has been shown to associate with presenilin *in vitro* through yeast-2-hyrbid assays [13] and natively through co-immunoprecipitation studies [34], we can speculate that such an interaction exists for KChIP2 and presenilin in the heart. Furthermore, it is not yet clear if this interaction serves to modulate the influence presenilin has over RyR activity, as previously shown in a heterologous system for KChIP3 [23], or if this interaction is also significant for mechanisms of presenilin trafficking and/or localization, as implicated in this investigation. Considerably, KChIP2 loss leads not only to the destabilization and relocalization of Kv4 channel expression but also shifts Kv4 channel kinetics, showing an established utilization of multimodal regulation as a consequence of KChIP2 interactions [4]. Therefore, it is entirely plausible that the redistribution of presenilin 1 expression and activity could be affected following KChIP2 loss.

While we show no change in the expression of a panel of calcium handling proteins, it is entirely possible that post-translational modifications to RyR may mediate the changes in CICR. In particular, phosphorylation of RyR can lead to a gain in CICR through an increase in open probability [35]. Therefore, a decline in the steady state level of RyR phosphorylation could be a relevant change consistent with this phenotype. However, phosphorylation of RyR has also been associated with an accelerated rate of SR Ca^2+^ release as well as slowed channel inactivation [36]. Here, in response to KChIP2 loss, we observe no change in the rate of Ca^2+^ release, reflected in our time-to-peak measurements (Figs 1D and 5C), while also producing significant delays in the decay rate for our Ca^2+^ transient and spark events (Figs 1C and 5D). The shifts we observe to RyR open probability and channel kinetics are therefore less likely to be compatible with the modifications contributed by a potentially reduced state of phosphorylation. At the same time, preliminary data using western blot evaluated two samples of guinea pig myocytes silenced for KChIP2, which revealed no change in phosphorylation status of RyR compared to control treated cells at the S2814 residue (data not shown). Therefore, it is unlikely that phosphorylation changes are responsible for the observed phenotype.

Overall, cardiac disease progression is defined largely by a continuing decline in cardiac performance. Investigations into the underlying phenotype has shown a multitude of gene changes and protein modifications can be responsible, including changes to *I*_Ca,L_, loss of SERCA2a expression and function leading to depletion of SR Ca^2+^ content, as well as modifications to the stability and activity of RyR Ca^2+^, influencing CICR response. Presenilin so far has only been implicated in hypertrophic remodeling associated with primary mutations [31], however, this establishes precedence that disruption to normal presenilin activity contributes to cardiac remodeling through its influence over RyR. Therefore, the relationship between KChIP2 loss and disruption of presenilin localization represents a novel pathway meaningful for acquired states of heart disease as well.

KChIP2 depletion continues to be one of the most consistent across multiple lines of pathogenesis. Critically, our data highlight the involvement of KChIP2 in contributing to the state of compromised cardiac output, establishing it as much more than a modifier of cardiac repolarization and depolarization. These contributions are critical when trying to understand the culmination of events leading to and worsening heart failure, where compromised contractility is paramount. Together, these data support KChIP2 as a potential new target for prevention of cardiac disease.

## Supporting information

S1 Table Individual data points contributing to the summary data for the Ca^2+^ transient amplitude (Fig 1B), Ca^2+^ transient duration at 50% peak (Fig 1C), and the Ca^2+^ transient time-to-peak (Fig 1D). (XLSX)

S2 Table Individual data points contributing to the summary data for fractional shortening (Fig 2B), duration to 50% relaxation (Fig 2C), and the time to peak contraction (Fig 2D). (XLSX)

S3 Table Individual data points contributing to the summary data for caffeine stimualtion to evaluate SR Ca^2+^ content (Fig 3B). (XLSX)

S4 Table Individual data points contributing to the summary data for immunoblot densitometry evaluating RyR, Serca2a, Pln, and NCX, normalized to GAPDH (Fig 4B). (XLSX)

S5 Table Individual data points contributing to the summary data for Ca^2+^ spark recordings, including spark frequency (Fig 5B), time-to-peak (Fig 5C), spark decay time (Fig 5D), and full width at half maximum (Fig 5E). (XLSX)

S6 Table Individual data points contributing to the summary data for presenilin 1 protein expression (Fig 6B), distribution of staining within regions of interest for presenilin 1 (Fig 6E) and RyR (Fig 6F), which also represents the averages and variance associated with those plots. (XLSX)

## Abbreviations

KChIP: K+ channel interacting protein
RyR: Ryanodine receptor
APD: Action Potential Duration
*I*_Ca,L_: L-type Ca^2+^ current
SERCA2a: SR Ca^2+^ ATPase
NCX: Sodium Calcium Exchanger
CICR: Calcium induced calcium release
IP_3_R: Inositol 1,4,5-trisphosphate receptor
NMDA: *N*-methyl-D-aspartate receptor
